# Aqua­bis(1*H*-imidazole-κ*N* ^3^)bis­(4-methyl­benzoato)-κ*O*;κ*O*,*O*′-nickel(II)

**DOI:** 10.1107/S1600536808009471

**Published:** 2008-04-10

**Authors:** Wen-Dong Song, Run-Zhen Fan, Hong-Mian Wu

**Affiliations:** a College of Science, Guang Dong Ocean University, Zhanjiang 524088, People’s Republic of China; b College of Food Science and Technology, Guang Dong Ocean University, Zhanjiang 524088, People’s Republic of China

## Abstract

In the mononuclear title compound, [Ni(C_8_H_7_O_2_)_2_(C_3_H_4_N_2_)_2_(H_2_O)], the Ni^II^ atom is coordinated by three carboxylate O atoms (from a bidentate 4-methyl­benzoate ligand and a monodentate 4-methyl­benzoate ligand), two N atoms (from two imidazole ligands) and a water mol­ecule in an octa­hedral geometry. Inter­molecular O—H⋯O hydrogen-bonding inter­actions lead to infinite chains, which are further self-assembled into a supra­molecular network through inter­molecular N—H⋯O hydrogen-bonding inter­actions and π–π stacking [centroid–centroid distance = 3.717 (2) Å].

## Related literature

For related literature, see: Song *et al.* (2007 ▶).
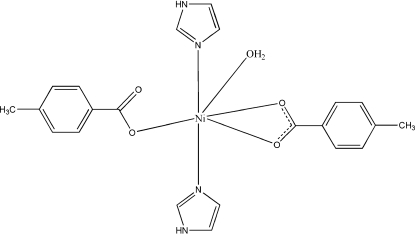


## Experimental

### 

#### Crystal data

[Ni(C_8_H_7_O_2_)_2_(C_3_H_4_N_2_)_2_(H_2_O)]
*M* *_r_* = 483.16
Monoclinic, 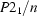

*a* = 18.9456 (12) Å
*b* = 5.8755 (4) Å
*c* = 20.3209 (14) Å
β = 101.813 (4)°
*V* = 2214.1 (3) Å^3^
*Z* = 4
Mo *K*α radiation
μ = 0.92 mm^−1^
*T* = 296 (2) K
0.30 × 0.26 × 0.25 mm

#### Data collection

Bruker APEXII area-detector diffractometer
Absorption correction: multi-scan (*SADABS*; Sheldrick, 1996 ▶) *T*_min_ = 0.770, *T*_max_ = 0.803
20580 measured reflections
3776 independent reflections
2815 reflections with *I* > 2σ(*I*)
*R*_int_ = 0.077

#### Refinement

*R*[*F* ^2^ > 2σ(*F* ^2^)] = 0.060
*wR*(*F* ^2^) = 0.171
*S* = 1.07
3776 reflections
297 parameters
3 restraints
H atoms treated by a mixture of independent and constrained refinement
Δρ_max_ = 1.02 e Å^−3^
Δρ_min_ = −0.71 e Å^−3^

### 

Data collection: *APEX2* (Bruker, 2004 ▶); cell refinement: *SAINT* (Bruker, 2004 ▶); data reduction: *SAINT*; program(s) used to solve structure: *SHELXS97* (Sheldrick, 2008 ▶); program(s) used to refine structure: *SHELXL97* (Sheldrick, 2008 ▶); molecular graphics: *XP* in *SHELXTL* (Sheldrick, 2008 ▶); software used to prepare material for publication: *SHELXTL*.

## Supplementary Material

Crystal structure: contains datablocks I, global. DOI: 10.1107/S1600536808009471/ng2442sup1.cif

Structure factors: contains datablocks I. DOI: 10.1107/S1600536808009471/ng2442Isup2.hkl

Additional supplementary materials:  crystallographic information; 3D view; checkCIF report

## Figures and Tables

**Table 1:** Hydrogen-bond geometry (Å, °)

*D*—H⋯*A*	*D*—H	H⋯*A*	*D*⋯*A*	*D*—H⋯*A*
O1*W*—H1*W*⋯O3^i^	0.826 (10)	2.31 (4)	2.782 (4)	116 (4)
O1*W*—H1*W*⋯O2^i^	0.826 (10)	2.21 (3)	2.772 (4)	126 (3)
N2—H2⋯O2^ii^	0.86	1.96	2.816 (5)	175
N4—H22⋯O4^iii^	0.86	2.02	2.865 (4)	167

Symmetry codes: (i) 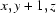; (ii) 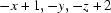; (iii) 
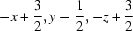
.

**Fig. 1.**
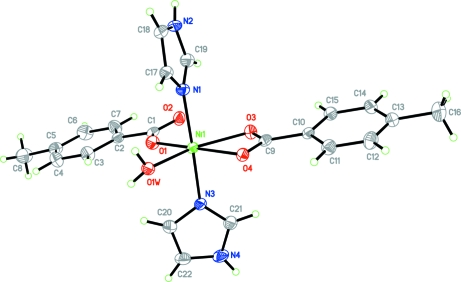
The structure of (I), showing the atomic numbering scheme. Non-H atoms are shown as 30% probability displacement ellipsoids.

**Fig. 2.**
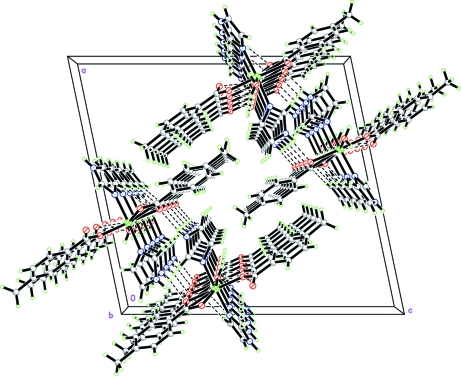
The packing of structure (I).

## supplementary crystallographic information

### Comment

In the structural investigation of 4-methylbenzate complexes, it has been found that the 4-methylbenzoic acid functions as a multidentate ligand [Song *et al.* (2007)], with versatile binding and coordination modes. In this paper, we report the crystal structure of the title compound, (I), a new Ni complex obtained by the reaction of 4-methylbenzoic acid, imidazole and nickel chloride in alkaline aqueous solution.

As illustrated in Figure 1, the Ni^II^ atom exists in a disordered octahedral environment, defined by three carboxyl O atoms from one bisdentate 4-methylbenzate ligand and one monodentate 4-methylbenzate ligand, two N atoms from two imidazole ligands and one water molecule. Intermolecular O—H···O hydrogen bonding interactions (Table 1) form infinite chains involving the coordinating water molecule as donors and O atoms of 4-methylbenzate ligands as acceptors, which are further self-assembled into a supramolecular network through intermolecular N—H···O hydrogen bonding interactions and π-π stacking interactions of neighboring complexes, with a centroid-centroid distance of 3.717 (2) Å. (Fig. 2).

### Experimental

A mixture of nickel chloride(1 mmol), 4-methylbenzoic acid (1 mmol), imidazole(1 mmol), NaOH (1.5 mmol) and H_2_O (12 ml) was placed in a 23 ml Teflon reactor, which was heated to 433 K for three days and then cooled to room temperature at a rate of 10 K h^-1^. The crystals obtained were washed with water and dryed in air.

### Refinement

H atoms were placed at calculated positions and were treated as riding on the parent C atoms with C—H = 0.93 - 0.97 Å, N—H = 0.86 Å, and with *U*_iso_(H) = 1.2 *U*_eq_(C, N).

### Crystal data

**Table:**

[Ni(C_8_H_7_O_2_)_2_(C_3_H_4_N_2_)_2_(H_2_O)]	*F*_000_ = 1008
*M**_r_* = 483.16	*D*_x_ = 1.449 Mg m^−^^3^
Monoclinic, *P*2_1_/*n*	Mo *K*α radiation λ = 0.71073 Å
Hall symbol: -P 2yn	Cell parameters from 3600 reflections
*a* = 18.9456 (12) Å	θ = 1.4–28º
*b* = 5.8755 (4) Å	µ = 0.92 mm^−^^1^
*c* = 20.3209 (14) Å	*T* = 296 (2) K
β = 101.813 (4)º	Block, blue
*V* = 2214.1 (3) Å^3^	0.30 × 0.26 × 0.25 mm
*Z* = 4	

### Data collection

**Table:**

Bruker APEXII area-detector diffractometer	3776 independent reflections
Radiation source: fine-focus sealed tube	2815 reflections with *I* > 2σ(*I*)
Monochromator: graphite	*R*_int_ = 0.077
*T* = 296(2) K	θ_max_ = 25.2º
φ and ω scans	θ_min_ = 1.7º
Absorption correction: multi-scan(SADABS; Sheldrick, 1996)	*h* = −22→22
*T*_min_ = 0.770, *T*_max_ = 0.803	*k* = −7→7
20580 measured reflections	*l* = −22→21

### Refinement

**Table:**

Refinement on *F*^2^	Secondary atom site location: difference Fourier map
Least-squares matrix: full	Hydrogen site location: inferred from neighbouring sites
*R*[*F*^2^ > 2σ(*F*^2^)] = 0.060	H atoms treated by a mixture of independent and constrained refinement
*wR*(*F*^2^) = 0.171	*w* = 1/[σ^2^(*F*_o_^2^) + (0.1064*P*)^2^ + 0.0659*P*] where *P* = (*F*_o_^2^ + 2*F*_c_^2^)/3
*S* = 1.07	(Δ/σ)_max_ = 0.001
3776 reflections	Δρ_max_ = 1.02 e Å^−^^3^
297 parameters	Δρ_min_ = −0.71 e Å^−^^3^
3 restraints	Extinction correction: none
Primary atom site location: structure-invariant direct methods	

### Special details

**Table:**

Geometry. All e.s.d.'s (except the e.s.d. in the dihedral angle between two l.s. planes) are estimated using the full covariance matrix. The cell e.s.d.'s are taken into account individually in the estimation of e.s.d.'s in distances, angles and torsion angles; correlations between e.s.d.'s in cell parameters are only used when they are defined by crystal symmetry. An approximate (isotropic) treatment of cell e.s.d.'s is used for estimating e.s.d.'s involving l.s. planes.
Refinement. Refinement of *F*^2^ against ALL reflections. The weighted *R*-factor *wR* and goodness of fit *S* are based on *F*^2^, conventional *R*-factors *R* are based on *F*, with *F* set to zero for negative *F*^2^. The threshold expression of *F*^2^ > σ(*F*^2^) is used only for calculating *R*-factors(gt) *etc*. and is not relevant to the choice of reflections for refinement. *R*-factors based on *F*^2^ are statistically about twice as large as those based on *F*, and *R*- factors based on ALL data will be even larger.

### Fractional atomic coordinates and isotropic or equivalent isotropic displacement parameters (Å^2^)

**Table:**

	*x*	*y*	*z*	*U*_iso_*/*U*_eq_	
C1	0.68032 (18)	0.0732 (7)	0.9997 (2)	0.0328 (10)	
C2	0.72717 (19)	0.1141 (7)	1.0678 (2)	0.0318 (10)	
C3	0.7618 (2)	0.3198 (8)	1.0827 (2)	0.0434 (12)	
H3	0.7552	0.4352	1.0507	0.052*	
C4	0.8066 (2)	0.3558 (9)	1.1451 (3)	0.0515 (13)	
H4	0.8302	0.4949	1.1538	0.062*	
C5	0.8171 (2)	0.1911 (9)	1.1943 (3)	0.0450 (12)	
C6	0.7816 (2)	−0.0143 (9)	1.1796 (3)	0.0532 (13)	
H6	0.7875	−0.1286	1.2119	0.064*	
C7	0.7377 (2)	−0.0515 (8)	1.1177 (3)	0.0473 (12)	
H7	0.7144	−0.1910	1.1090	0.057*	
C8	0.8676 (3)	0.2326 (11)	1.2602 (3)	0.0676 (17)	
H8A	0.9136	0.1655	1.2593	0.101*	
H8B	0.8482	0.1655	1.2957	0.101*	
H8C	0.8733	0.3935	1.2677	0.101*	
C9	0.56235 (18)	0.1063 (7)	0.7571 (2)	0.0291 (9)	
C10	0.51866 (17)	0.0039 (6)	0.6958 (2)	0.0282 (9)	
C11	0.5069 (2)	0.1198 (8)	0.6345 (2)	0.0402 (11)	
H11	0.5271	0.2633	0.6325	0.048*	
C12	0.4665 (2)	0.0269 (8)	0.5775 (2)	0.0463 (12)	
H12	0.4593	0.1088	0.5375	0.056*	
C13	0.4360 (2)	−0.1874 (8)	0.5782 (3)	0.0442 (12)	
C14	0.4474 (2)	−0.3059 (8)	0.6384 (3)	0.0406 (12)	
H14	0.4271	−0.4496	0.6398	0.049*	
C15	0.4884 (2)	−0.2135 (7)	0.6964 (2)	0.0351 (11)	
H15	0.4960	−0.2964	0.7362	0.042*	
C16	0.3904 (3)	−0.2924 (10)	0.5148 (3)	0.0693 (17)	
H16A	0.4187	−0.3031	0.4807	0.104*	
H16B	0.3490	−0.1985	0.4989	0.104*	
H16C	0.3750	−0.4417	0.5249	0.104*	
C17	0.49535 (19)	0.5721 (7)	0.8861 (2)	0.0373 (11)	
H17	0.5079	0.7042	0.8660	0.045*	
C18	0.4381 (2)	0.5510 (8)	0.9164 (2)	0.0433 (12)	
H18	0.4047	0.6627	0.9210	0.052*	
C19	0.4959 (2)	0.2304 (8)	0.9211 (3)	0.0411 (12)	
H19	0.5083	0.0787	0.9301	0.049*	
C20	0.7912 (2)	0.2900 (8)	0.8792 (3)	0.0495 (14)	
H20	0.7947	0.3948	0.9140	0.059*	
C21	0.7474 (2)	0.0663 (7)	0.7991 (2)	0.0420 (11)	
H21	0.7152	−0.0160	0.7671	0.050*	
N4	0.81940 (17)	0.0528 (6)	0.8074 (2)	0.0467 (10)	
H22	0.8430	−0.0306	0.7846	0.056*	
N1	0.53185 (15)	0.3705 (6)	0.88955 (18)	0.0333 (8)	
N2	0.43972 (17)	0.3329 (7)	0.93841 (19)	0.0413 (10)	
H2	0.4098	0.2716	0.9598	0.050*	
N3	0.72811 (16)	0.2084 (5)	0.84118 (19)	0.0322 (9)	
C22	0.8477 (2)	0.1935 (9)	0.8579 (3)	0.0499 (14)	
H4A	0.8964	0.2199	0.8749	0.060*	
Ni1	0.62851 (2)	0.29313 (8)	0.86285 (3)	0.0263 (2)	
O1	0.67349 (15)	0.2409 (5)	0.95998 (16)	0.0380 (8)	
O2	0.65064 (15)	−0.1153 (5)	0.98695 (15)	0.0437 (8)	
O3	0.57902 (13)	−0.0107 (4)	0.80995 (15)	0.0338 (7)	
O4	0.58228 (13)	0.3134 (4)	0.75769 (15)	0.0300 (7)	
O1W	0.66137 (13)	0.6312 (5)	0.87438 (15)	0.0344 (7)	
H1W	0.6334 (14)	0.721 (6)	0.887 (2)	0.052*	
H2W	0.7005 (9)	0.663 (7)	0.896 (2)	0.052*	

### Atomic displacement parameters (Å^2^)

**Table:**

	*U*^11^	*U*^22^	*U*^33^	*U*^12^	*U*^13^	*U*^23^
C1	0.0320 (19)	0.036 (2)	0.034 (3)	0.0060 (16)	0.0155 (18)	−0.002 (2)
C2	0.0314 (18)	0.038 (2)	0.027 (3)	0.0003 (17)	0.0100 (17)	0.001 (2)
C3	0.049 (2)	0.042 (3)	0.036 (3)	−0.0022 (19)	0.002 (2)	0.009 (2)
C4	0.047 (2)	0.053 (3)	0.050 (4)	−0.008 (2)	0.001 (2)	−0.005 (3)
C5	0.035 (2)	0.069 (3)	0.029 (3)	0.005 (2)	0.0035 (19)	0.002 (3)
C6	0.054 (3)	0.068 (3)	0.035 (3)	0.002 (2)	0.001 (2)	0.021 (3)
C7	0.048 (2)	0.047 (3)	0.046 (3)	−0.007 (2)	0.006 (2)	0.004 (2)
C8	0.050 (3)	0.107 (5)	0.043 (4)	0.002 (3)	0.001 (3)	0.001 (3)
C9	0.0326 (18)	0.026 (2)	0.031 (3)	0.0049 (16)	0.0122 (17)	0.001 (2)
C10	0.0306 (18)	0.030 (2)	0.026 (3)	0.0001 (15)	0.0104 (16)	0.0001 (19)
C11	0.046 (2)	0.034 (2)	0.041 (3)	−0.0007 (19)	0.011 (2)	−0.002 (2)
C12	0.057 (3)	0.048 (3)	0.032 (3)	0.003 (2)	0.005 (2)	0.004 (2)
C13	0.036 (2)	0.055 (3)	0.042 (3)	−0.0048 (19)	0.008 (2)	−0.012 (3)
C14	0.038 (2)	0.040 (3)	0.044 (4)	−0.0082 (17)	0.008 (2)	−0.008 (2)
C15	0.039 (2)	0.030 (2)	0.038 (3)	−0.0015 (16)	0.0107 (19)	−0.001 (2)
C16	0.070 (3)	0.079 (4)	0.057 (5)	−0.020 (3)	0.008 (3)	−0.025 (3)
C17	0.036 (2)	0.034 (2)	0.044 (3)	−0.0018 (16)	0.0125 (18)	−0.007 (2)
C18	0.036 (2)	0.052 (3)	0.045 (3)	0.0025 (19)	0.014 (2)	−0.013 (2)
C19	0.040 (2)	0.042 (3)	0.043 (3)	−0.0010 (18)	0.010 (2)	0.000 (2)
C20	0.034 (2)	0.060 (3)	0.056 (4)	−0.0034 (19)	0.013 (2)	−0.016 (3)
C21	0.043 (2)	0.036 (2)	0.049 (3)	0.0004 (18)	0.015 (2)	−0.008 (2)
N4	0.0425 (19)	0.045 (2)	0.059 (3)	0.0097 (16)	0.0251 (18)	−0.005 (2)
N1	0.0318 (16)	0.0358 (19)	0.035 (2)	−0.0025 (14)	0.0123 (14)	0.0007 (17)
N2	0.0344 (17)	0.058 (3)	0.037 (3)	−0.0090 (16)	0.0194 (16)	−0.006 (2)
N3	0.0329 (16)	0.0297 (18)	0.036 (3)	0.0001 (13)	0.0130 (15)	−0.0062 (17)
C22	0.034 (2)	0.064 (3)	0.055 (4)	0.000 (2)	0.016 (2)	−0.001 (3)
Ni1	0.0288 (3)	0.0243 (3)	0.0276 (4)	0.00023 (17)	0.0098 (2)	0.0002 (2)
O1	0.0404 (15)	0.0427 (18)	0.030 (2)	0.0005 (12)	0.0042 (13)	0.0050 (14)
O2	0.0580 (17)	0.0408 (18)	0.035 (2)	−0.0158 (15)	0.0168 (14)	−0.0062 (16)
O3	0.0400 (14)	0.0276 (15)	0.032 (2)	−0.0014 (11)	0.0030 (12)	0.0030 (14)
O4	0.0374 (14)	0.0249 (15)	0.0286 (19)	−0.0026 (11)	0.0090 (12)	0.0007 (13)
O1W	0.0316 (13)	0.0292 (15)	0.044 (2)	−0.0014 (12)	0.0124 (13)	−0.0067 (14)

### Geometric parameters (Å, °)

**Table:**

C1—O2	1.245 (5)	C15—H15	0.9300
C1—O1	1.264 (5)	C16—H16A	0.9600
C1—C2	1.503 (6)	C16—H16B	0.9600
C2—C3	1.379 (6)	C16—H16C	0.9600
C2—C7	1.389 (6)	C17—C18	1.359 (6)
C3—C4	1.391 (6)	C17—N1	1.366 (5)
C3—H3	0.9300	C17—H17	0.9300
C4—C5	1.376 (7)	C18—N2	1.356 (6)
C4—H4	0.9300	C18—H18	0.9300
C5—C6	1.384 (7)	C19—N1	1.315 (5)
C5—C8	1.497 (7)	C19—N2	1.333 (5)
C6—C7	1.376 (6)	C19—H19	0.9300
C6—H6	0.9300	C20—C22	1.359 (6)
C7—H7	0.9300	C20—N3	1.370 (5)
C8—H8A	0.9600	C20—H20	0.9300
C8—H8B	0.9600	C21—N3	1.300 (5)
C8—H8C	0.9600	C21—N4	1.342 (5)
C9—O3	1.260 (5)	C21—H21	0.9300
C9—O4	1.274 (5)	N4—C22	1.341 (6)
C9—C10	1.475 (6)	N4—H22	0.8600
C10—C11	1.396 (6)	N1—Ni1	2.065 (3)
C10—C15	1.401 (5)	N2—H2	0.8600
C11—C12	1.365 (6)	N3—Ni1	2.084 (3)
C11—H11	0.9300	C22—H4A	0.9300
C12—C13	1.387 (6)	Ni1—O1	2.007 (3)
C12—H12	0.9300	Ni1—O1W	2.081 (3)
C13—C14	1.385 (7)	Ni1—O4	2.140 (3)
C13—C16	1.526 (7)	Ni1—O3	2.193 (3)
C14—C15	1.383 (6)	O1W—H1W	0.826 (10)
C14—H14	0.9300	O1W—H2W	0.805 (10)
			
O2—C1—O1	125.3 (4)	H16B—C16—H16C	109.5
O2—C1—C2	119.4 (4)	C18—C17—N1	109.9 (4)
O1—C1—C2	115.3 (4)	C18—C17—H17	125.1
C3—C2—C7	117.6 (4)	N1—C17—H17	125.1
C3—C2—C1	120.4 (4)	N2—C18—C17	105.6 (4)
C7—C2—C1	121.9 (4)	N2—C18—H18	127.2
C2—C3—C4	120.5 (4)	C17—C18—H18	127.2
C2—C3—H3	119.8	N1—C19—N2	111.5 (4)
C4—C3—H3	119.8	N1—C19—H19	124.2
C5—C4—C3	121.7 (5)	N2—C19—H19	124.2
C5—C4—H4	119.1	C22—C20—N3	109.1 (4)
C3—C4—H4	119.1	C22—C20—H20	125.4
C4—C5—C6	117.7 (4)	N3—C20—H20	125.4
C4—C5—C8	120.3 (5)	N3—C21—N4	111.7 (4)
C6—C5—C8	121.9 (5)	N3—C21—H21	124.1
C7—C6—C5	120.8 (5)	N4—C21—H21	124.1
C7—C6—H6	119.6	C22—N4—C21	107.3 (4)
C5—C6—H6	119.6	C22—N4—H22	126.4
C6—C7—C2	121.7 (4)	C21—N4—H22	126.4
C6—C7—H7	119.2	C19—N1—C17	105.1 (3)
C2—C7—H7	119.2	C19—N1—Ni1	124.4 (3)
C5—C8—H8A	109.5	C17—N1—Ni1	130.1 (3)
C5—C8—H8B	109.5	C19—N2—C18	107.8 (4)
H8A—C8—H8B	109.5	C19—N2—H2	126.1
C5—C8—H8C	109.5	C18—N2—H2	126.1
H8A—C8—H8C	109.5	C21—N3—C20	105.4 (3)
H8B—C8—H8C	109.5	C21—N3—Ni1	132.9 (3)
O3—C9—O4	119.4 (4)	C20—N3—Ni1	121.3 (3)
O3—C9—C10	119.8 (4)	N4—C22—C20	106.5 (4)
O4—C9—C10	120.8 (4)	N4—C22—H4A	126.8
C11—C10—C15	117.5 (4)	C20—C22—H4A	126.8
C11—C10—C9	120.9 (4)	O1—Ni1—N1	89.78 (13)
C15—C10—C9	121.6 (4)	O1—Ni1—O1W	88.76 (12)
C12—C11—C10	121.4 (4)	N1—Ni1—O1W	91.21 (12)
C12—C11—H11	119.3	O1—Ni1—N3	87.18 (13)
C10—C11—H11	119.3	N1—Ni1—N3	176.89 (14)
C11—C12—C13	121.1 (5)	O1W—Ni1—N3	89.37 (11)
C11—C12—H12	119.4	O1—Ni1—O4	174.26 (11)
C13—C12—H12	119.4	N1—Ni1—O4	92.66 (12)
C14—C13—C12	118.4 (4)	O1W—Ni1—O4	96.38 (11)
C14—C13—C16	120.1 (5)	N3—Ni1—O4	90.32 (12)
C12—C13—C16	121.5 (5)	O1—Ni1—O3	114.23 (12)
C15—C14—C13	120.9 (4)	N1—Ni1—O3	89.70 (12)
C15—C14—H14	119.5	O1W—Ni1—O3	157.00 (12)
C13—C14—H14	119.5	N3—Ni1—O3	90.96 (11)
C14—C15—C10	120.6 (4)	O4—Ni1—O3	60.62 (10)
C14—C15—H15	119.7	C1—O1—Ni1	135.7 (3)
C10—C15—H15	119.7	C9—O3—Ni1	88.9 (2)
C13—C16—H16A	109.5	C9—O4—Ni1	90.9 (2)
C13—C16—H16B	109.5	Ni1—O1W—H1W	116 (3)
H16A—C16—H16B	109.5	Ni1—O1W—H2W	120 (3)
C13—C16—H16C	109.5	H1W—O1W—H2W	104.8 (17)
H16A—C16—H16C	109.5		

### Hydrogen-bond geometry (Å, °)

**Table:**

*D*—H···*A*	*D*—H	H···*A*	*D*···*A*	*D*—H···*A*
O1W—H1W···O3^i^	0.826 (10)	2.31 (4)	2.782 (4)	116 (4)
O1W—H1W···O2^i^	0.826 (10)	2.21 (3)	2.772 (4)	126 (3)
N2—H2···O2^ii^	0.86	1.96	2.816 (5)	175
N4—H22···O4^iii^	0.86	2.02	2.865 (4)	167

Symmetry codes: (i) *x*, *y*+1, *z*; (ii) −*x*+1, −*y*, −*z*+2; (iii) −*x*+3/2, *y*−1/2, −*z*+3/2.
